# Toward data lakes as central building blocks for data management and analysis

**DOI:** 10.3389/fdata.2022.945720

**Published:** 2022-08-19

**Authors:** Philipp Wieder, Hendrik Nolte

**Affiliations:** Gesellschaft für wissenschaftliche Datenverarbeitung mbH Göttingen (GWDG), Göttingen, Germany

**Keywords:** data lake, data analytics, research data management, provenance, big data, FAIR

## Abstract

Data lakes are a fundamental building block for many industrial data analysis solutions and becoming increasingly popular in research. Often associated with big data use cases, data lakes are, for example, used as central data management systems of research institutions or as the core entity of machine learning pipelines. The basic underlying idea of retaining data in its native format within a data lake facilitates a large range of use cases and improves data reusability, especially when compared to the schema-on-write approach applied in data warehouses, where data is transformed prior to the actual storage to fit a predefined schema. Storing such massive amounts of raw data, however, has its very own challenges, spanning from the general data modeling, and indexing for concise querying to the integration of suitable and scalable compute capabilities. In this contribution, influential papers of the last decade have been selected to provide a comprehensive overview of developments and obtained results. The papers are analyzed with regard to the applicability of their input to data lakes that serve as central data management systems of research institutions. To achieve this, contributions to data lake architectures, metadata models, data provenance, workflow support, and FAIR principles are investigated. Last, but not least, these capabilities are mapped onto the requirements of two common research personae to identify open challenges. With that, potential research topics are determined, which have to be tackled toward the applicability of data lakes as central building blocks for research data management.

## 1. Introduction

In recent years, data lakes have become increasingly popular in various industrial and academic domains. In particular for academia, data lakes come with the promise to provide solutions for several data management challenges at once. Similar to *Data Warehouses* (Devlin and Murphy, 1988; Inmon, 2005), data lakes aim at integrating heterogeneous data from different sources into a single, homogeneous data management system. This allows data holders to overcome the limits of disparate and isolated data silos and enforce uniform data governance.

Data Warehouses have a fixed schema, which implies a so-called *schema-on-write* approach to feed data into them. *Extract-Transform-Load* (ETL) processes are therefore needed to extract the raw data from its source, transform, e.g., to clean it or to fit it into the predefined schema, and then load it into the Data Warehouse (El-Sappagh et al., 2011). Although there are some known challenges when using these ETL processes (Munappy et al., 2020), the main drawback is the loss of information during the transformation to fit data into the fixed schema. To prevent this information loss, which limits the reuse of the data e.g., for research questions outside the original scope, James Dixon proposed the data lake concept in Dixon (2010). Here, in contrast to the schema-on-write approach of a Data Warehouses, data is retained in its original format and a schema is only inferred when a subsequent process reads the data, an approach which is termed *schema-on-read*.

The necessity for low cost and highly scalable mass storage with the ability to be integrated into parallelised computations was recognized as a key feature already at the advent of data lakes, leading to a close connection between a data lakes and *Apache Hadoop* (Khine and Wang, 2018). This approach was at some point challenged by large cloud providers like Amazon or Microsoft and their proprietary data lake solutions like *AWS Lake Formation* or *Azure Data Lake* (Hukkeri et al., 2020; Aundhkar and Guja, 2021). These products introduced, among other features, the separation of storage and compute and offered customers the well-known cloud features like the pay-as-you-go payment model.

Although a data lake implements a schema-on-read semantic, some modeling is mandatory to ensure proper data integration, comprehensibility, and quality (Hai et al., 2018). Such data modeling typically consists of a conceptual model, which should facilitate frequent changes and therefore should not enforce a fixed schema (Mathis, 2017; Khine and Wang, 2018). The required metadata can be gathered by extracting prescriptive information from the data itself, for instance by reading out header information, or metadata can be additionally extracted from the source along with the original raw data itself. In addition, data can be continuously enriched with metadata during its lifetime in the data lake, for instance by identifying relationships among the different data sets (Hai et al., 2016; Sawadogo et al., 2019) or by auditing provenance information.

Quite some literature exists related to the use of data lakes in different industries (Terrizzano et al., 2015; Golec, 2019; Hukkeri et al., 2020), particularly with the intent to manage big amounts of data (Miloslavskaya and Tolstoy, 2016). However, there is also huge potential for the adoption of data lakes in research institutions. One benefit is, for example, that data silos, which quickly arise when different research teams work independently, can be prevented or integrated. This also enables novel analysis approaches across an homogeneous data set, which are not possible dealing with distributed and isolated data silos. Another advantage is that a common data governance can be enforced on an overarching level, like an institute or a research project, to guarantee a predefined data quality level while and to assist researchers to adhere to good scientific practices.

When scaling out the usage of a data lake across an entire research institution to be the central research data management system, one is encountered with different use cases and users who have a diverse skill set. In this paper we want to explore the current state of the art of data lakes and make based on this survey an applicability analysis of the presented works in the context of a large research institution. For this, papers were collected, which had unique contributions to at least one of the topics presented below. We start in Section 2 with a discussion about the existing Data Lake Architectures, which offers an overview about the highest level of organization and abstraction of a given implementation. In the following Section 3 different metadata models are presented, which lie conceptually one layer below the general architecture, and ensure the correct data organization in the data lake, which involves semantic information about the data itself as well as metadata describing the relationships among the data. One if not the most important relationship which needs to be modeled in a data lake is the data lineage, which is in detail discussed in Section 4. Closely related to the topic of provenance auditing is the general ability to perform automated data analytics workflows, ideally in a scalable manner, on the data lake, which is discussed in the following Section 5. In Section 6.2 two disparate data lake users, i.e., a domain researcher and a data scientist, are used to perform an applicability analysis of the before presented works. In addition a comparison based on common but also topic-specific criteria is done to extend on the generic applicability analysis. Based on the general survey in each of these topics, future challenges are identified.

## 2. Data lake architectures

As of today, a lot of development and analysis was conducted in the area of *data lake architectures*, where the so-called *zone architecture* (Patel et al., 2017; Ravat and Zhao, 2019), including the *pond architecture* (Inmon, 2016), became the most cited and used. These architectures have already been surveyed by Hai et al. (2021) and Sawadogo and Darmont (2021), and a *functional* architecture was proposed by both of them, and a *maturity* and a *hybrid* architecture have been derived by Sawadogo and Darmont (2021). These surveys, however, did not include recent works like the definition of a zone reference model (Giebler et al., 2020) or a data lake architecture based on *FAIR Digital Objects* (FDOs) (Nolte and Wieder, 2022).

### 2.1. Definition of the term data lake architecture

The term data lake architecture was defined by Giebler et al. (2021) to represent the comprehensive design of a data lake, including the infrastructure, data storage, data flow, data modeling, data organization, data processes, metadata management, data security and privacy, and data quality. In this data lake architecture framework, only the data security and privacy and the data quality are considered to be purely conceptual, whereas the other aspects include a conceptual and a physical, i.e., system specific, dimension. As stated by Madera and Laurent (2016) more generically, a data lake generally has a logical and a physical organization. In this paper, we refer to the term *data lake architecture* only with respect to the conceptual organization of a data lake in the highest level of abstraction, since this should make this work more comparable to the existing literature, although there exists a strong dependency on other aspects of a data lake, like the metadata modeling.

### 2.2. Zone architecture

The general idea to divide a data lake into different zones arises from the necessity to automatically run standardized pre-processing pipelines, organize the resulting pre-processed data, and make it available to subsequent processing steps, like reporting, *Online Analytical Processing* (OLAP), and particularly advanced analytics. This is achieved by assigning data to different zones based on the degree of processing, and sometimes the intended future use case. Therefore, it is common to have a *raw data zone*, where, according to the original idea of a data lake, data is retained in its raw format to facilitate repetition of processes or the application of new methods based on the original data. Pre-processed data is then usually collected in a dedicated zone for *pre-processed*, or *refined* data, sometimes called *staging zone* (Zikopoulos, 2015) or *processing zone* (Ravat and Zhao, 2019). Data that requires additional governance can be collected in a dedicated zone of its own called *trusted zone* (Zikopoulos, 2015), or *sensitive zone* (Gorelik, 2019).

The most extensive analysis of the *zone architecture* was conducted by Giebler et al. (2020), where five different data lakes based on the *zone architecture* (Madsen, 2015; Zikopoulos, 2015; Patel et al., 2017; Sharma, 2018; Gorelik, 2019; Ravat and Zhao, 2019) were analyzed with respect to their design differences, specific features and their individual use cases in order to derive a generic meta-model for a zone, and to specify a zone reference model based on it. Giebler et al. identified that a zone is uniquely defined by the characteristics of the data contained in that zone, the intrinsic properties a zone enforces on the data, the user groups which are intended to work in that zone, the modeling approach to organize the corresponding metadata, and the data sources as well as destinations. In the presented zone reference model, Giebler et al. propose to split the zones up in a *raw zone* and a *harmonized zone*, which is use case independent, and a use case-specific *distilled zone*, which serves data to the final *delivery zone*, to support reporting and OLAP tasks, and a *explorative zone* to support advanced analytics. Each zone hosts a *protected* area for data that requires special governance. The actual implementation and the deployed systems can vary in each zone, including the storage, the metadata model, and the metadata management system itself. This entails synchronously, that also the user interface potentially changes with each zone.

### 2.3. Lambda architecture

The *Lambda Architecture* has been proposed to enhance the capability of a data lake to process data streams in near real-time instead of fully ingesting hot data into the data lake and performing batch-processing with a certain time delay (Mathis, 2017). However, retaining all raw data in its native format is the core idea of a data lake. In order to resolve this contradiction, the Lambda Architecture (Warren and Marz, 2015) implements two processing streams in parallel. Here, data is being processed in near real-time in the *speed layer*, whereas the *batch layer* ingests data into the data lake and performs some predefined processing steps. There have been numerous implementations proposed for a data lake utilizing the Lambda Architecture (Hasani et al., 2014; Villari et al., 2014; Batyuk and Voityshyn, 2016). However, the following two particular works are presented which are building on top of public cloud offerings.

A Lambda Architecture was used by Munshi and Mohamed (2018) to build a data lake for smart grid data analytics using Google's cloud computing as Infrastructure as a Service (IaaS). Here, the data is collected by a dedicated *Data Collecting Layer*, in this particular case realized by Apache *Flume*^1^. From there, the data is sent to the core of this specific data lake implementation, a Hadoop Cluster. The master node stores the data on HDFS (Borthakur, 2007), and computes arbitrary, predefined functions using MapReduce (Dean and Ghemawat, 2008). The speed layer is implemented using *Apache Spark* (Zaharia et al., 2010). The serving layer combines the output of the batch and the speed layer and provides a batch view of the relevant data, using e.g., *Hive* (Thusoo et al., 2009), *Impala* as shown by Li (2014), and *Spark SQL* (Armbrust et al., 2015).

Similarly, Pérez-Arteaga et al. (2018) compared three different implementations using the Software as a Service (SaaS) offerings with a focus on serverless delivery of three different public cloud providers, i.e., Google Cloud Platform, Microsoft Azure, and Amazon Web Services Cloud. On AWS the speed layer accepts data *via* Kinesis Data streams and processes them using Kinesis Analytics and AWS Lambda. The results are stored in a dedicated *S3-Speed-Bucket*. Similarly, the batch layer uses Kinesis Firehose to ingest data into AWS Lambda, from where it is stored in an *S3-Batch-Bucket*. From here the data is read by AWS Glue and stored in an *S3-Result-Bucket*. The serving layer is realized by Athena which reads the data from both, the *S3-Result-Bucket* and the *S3-Speed-Bucket*. In the Google Cloud implementation, data is ingested by Pub/Sub to the speed and the batch layer, which are both realized using Dataflow. On the batch layer, an additional Datastore is employed to retain the raw incoming datasets. The serving layer uses BigQuery. On Microsoft Azure, data is received and ingested by EventHub. The speed layer uses Stream Analytics and forwards directly into the Serving Layer which is Cosmos DB. The batch layer also uses Stream Analytics to store the raw data into Data Lake Store (Ramakrishnan et al., 2017). From there it is read by Data lake Analytics, which also stores its results in Cosmos DB.

### 2.4. Lakehouse

*Lakehouses* as described by Armbrust et al. (2021) are a consequence of the general observation that in some cases the raw data from a data lake is used as an input for an ETL process to populate a data warehouse. The first step into a more unified setup was provided by *Delta lakes* (Armbrust et al., 2020), which provides ACID (atomicity, consistency, isolation, durability) transactions on cloud object storage for tables stores. These tables can be accessed from different systems, like Spark, Hive, Presto (Sethi et al., 2019), and others. This approach introduces, among other things, the advantage of still separating storage and compute. Lakehouses offer on top of ACID transactions direct access to the storage with traditional database semantics, e.g., SQL, using open file formats like *Apache Parquet* (Vohra, 2016) or *ORC* ^2^. Therefore, a metadata layer on top of the cloud storage can provide convenient SQL-like access to tables, while compute-intensive, non-SQL code, like machine learning, can directly access the files on the storage devices and thereby get higher performance.

### 2.5. FAIR digital object-based architecture

Using a *FAIR Digital Object*-based architecture, as proposed by Nolte and Wieder (2022), the data lake is not divided into different zones but realizes a flat, homogeneous, and uniform research data management from the user's point of view. To allow for segregation of data with a different pedigree of subjected processing, the *FAIR Digital Object* encapsulating the corresponding data has a certain type with which the delimitation between different data points is represented. This can mean in a simple example, that in practice there is a *Scanner*−*X*_*Raw*_ data type and a *Scanner*−*X*_*Preprocessed*_ data type. This leads to a much more fine-grained partition of the data lake as compared to the *zone-architecture*. This highly segregated data lake, however, does not entail a correlated increase in system complexity and administrative effort, since only one, or a necessary subset of types of the *FAIR Digital Objects* needs to be implemented and can then be further inherited from, hereby reusing the existing implementation on the used infrastructure. Since everything in this data lake is a *FAIR Digital Object*, not only data but also including workflows and execution environments, the user interface is completely homogeneous, since the user interacts with these objects by calling predefined functions. Each of these data objects is equivalently suited as input for automated workflows or user-defined advanced analytics. The requirement for additional governance or security measures can be defined on a per object basis and can be globally enforced based on the typed attributes describing the metadata of the *FAIR Digital Object*.

### 2.6. Functional and maturity-based architectures

The classification into *functional* and *maturity*-oriented data lake architectures do, unlike in the case of the zone, lambda, lakehouse, and FAIR Digital Object-based architectures, not represent yet another design concept, but rather serve as an improved way for classifying the different architectural approaches. The goal is to allow for a more modular comparison of existing data lake solutions and to better plan, the data life-cycle as well as to help match the individual functionality of the architectural pieces, which are building up the data lake, like zones, or objects, on the required infrastructure.

Within a *functional*-based architecture classification, the data lake is analyzed toward its operations which are performed on the data while moving through the general data lake workflow. Hai et al. (2021) define three layers, *ingestion, maintenance*, and *exploration*, where corresponding functions are then sub-grouped. A similar definition is provided by Sawadogo and Darmont (2021), where the four main components of a data lake are defined as *ingestion, storage, processing, querying*.

Following the maturity-based architecture classification, the degree of the processing of the data is the central point of consideration. This classification is only helpful in the discrimination and organization of different data sets, however, it completely lacks consideration of workflows and processing capabilities. However, Sawadogo and Darmont (2021) highlight the advantage of the planning of the data life-cycle. Therefore, a *hybrid architecture* was proposed by Sawadogo and Darmont (2021) alongside the *functional* and *maturity* based classifications. Within this architecture, the individual components are uniquely identified by the data refinement and the possible functionality, that can be performed on the specific set of data.

## 3. Metadata models

Proper metadata management is key to prevent that a data lake turns into a data swamp and thus is the most important component to ensure a continuous operation and usability (Walker and Alrehamy, 2015; Khine and Wang, 2018). Due to the generally flat hierarchy and the requirement to store any data in its native format, there is always the risk of losing the overall comprehensibility of the data lake. This comprehensibility is lost, if data cannot be found or the relationship to other data sets cannot be retrieved. One of the most severe consequences of this is the inability to define concise queries to select the data one is looking for in a fine-grained manner. As a consequence, numerous metadata models and systems tailor-made for usage in data lakes have been proposed. These models and systems originate from different use cases and represent various viewpoints, and therefore differ regarding their feature sets. From this wide variety of available options, a few distinct works have been selected and are discussed in the following sections.

### 3.1. Data vault

Data modeling in a *data vault* was proposed by Linstedt in the 1990s and published in the 2000s to allow for a more agile metadata evolution, i.e., the continuous development of the metadata schema, in data warehouses, compared to star or a snowflake schemata (Lindstedt and Graziano, 2011). This ensemble modeling uses traditionally relational database systems and combines the third normal form with the star schema. All data is stored in three different types of tables. *Hubs* describe a business concept and are implemented as lists of unique keys, and can be populated by different data sources. *Links* describe relationships between the aforementioned hubs. *Satellites* contain all attributes which describe the properties of a hub or a link. Evolving the data vault over time then mainly implies adding additional satellite tables to links and hubs. Therefore, there is no need to migrate existing tables, which facilities the continuous addition of metadata over time.

Due to these characteristics of the data vault concept, it was also applied in data lakes. Nogueira et al. (2018) explained the definition of a data vault for a specific use case and discussed the advantages of porting it to a NoSQL database by comparing benchmark results compared to a SQL database. They also exemplify how new data sources can be added by defining new hubs, links, and particularly satellites. Giebler et al. (2019) proposed to split the one central, data lake-wide data vault up into three distinct sub-data vaults: the *Raw Vault*, the *Business Vault*, and the *Data Mart*, whereby the latter does not necessarily need to be modeled in a data vault, but could also be a flat table, or a star schema. The authors reported that the agile approach along with the ability to make incremental updates serves well the needs for a data lake implementation. However, they pointed out that it can be hard to enforce business rules across the independent sub-data vaults, which they use, that managing ambiguous keys can not finally be solved, and that high-frequency data can critically inflate satellites.

### 3.2. GEMMS

*GEMMS* is proposed by Quix et al. (2016) as a *Generic and Extensible Metadata Management System* with a particular focus on scientific data management and, in this context, specifically for the domain of live sciences. The key component of GEMMS is an abstract entity called *Data Unit*, which consists of raw data and its associated metadata. It is stated, that the main advantages are, flexibility during ingestion and a user interface that abstracts singular files. These *Data Units* can be annotated with semantic metadata according to a suitable ontology. However, the core is described with *structure metadata*. Mappings are only discussed for semi-structured files, like CSV, XML, or spreadsheets, however, it seems straightforward to extend this in other use cases.

### 3.3. MEDAL and goldMEDAL

A graph-based metadata model was presented by Sawadogo et al. (2019), where a subset of data, called an object, is represented as a hypernode that contains all information about that particular object, like the version, semantic information, or something called *representations*. *Representations* present the data in a specific way, for instance as a word cloud for textual data. There is at least one representation required per object, which is connected to this object by a *Transformation*. These representations can be *transformed*, which is represented as a directed edge in the hypergraph. This edge contains information about the *transformation*, i.e., a script description or similar. Data versioning is performed at the attribute level of these hyperedges connecting two different representations. Additionally, it is possible to define undetected hyperedges representing the similarity of two objects, provided that the two data sets are comparable.

This approach was revised by Scholly et al. (2021). Here, the concept was simplified to only use *data entities, processes, links*, and *groupings*. *Processes* also generate new *data entities*, dropping the rather complicated idea of *representations*. These concepts are again mapped on a hypergraph. Both models require global metadata, such as *ontologies* or *thesauri*.

### 3.4. CODAL

The data lake, and in particular the utilized metadata model called *CODAL* (Sawadogo et al., 2019) was purpose-built for textual data. It combines a graph model connecting all ingested data sets with a data vault describing an individual data set. One core component is the *xml manifest*, which is divided into three parts: i) *atomic metadata*, ii) *non-atomic metadata*, and iii) a division for *physical relational metadata*. Metadata of the first category can be described as key-value pairs, whereas *non-atomic metadata* only contain references to a specific entity on a different system, they are "stored in a specific format in the filesystem" (Sawadogo et al., 2019). Additional information about the link strength which is modeling the *relational metadata* is stored in a dedicated graph database. Here, each node represents one document with a reference to the corresponding *xml manifest*.

### 3.5. Network-based models

A network-based model, which extends the simple categorization by Oram (2015) into three distinct types of metadata, i.e., *Business Metadata, Operational Metadata*, and *Technical Metadata*, was proposed by Diamantini et al. (2018) to improve the data integration of different data sources ingesting heterogeneous and unstructured data into the data lake. Here, the notion of objects, or nodes in the resulting graph, are used as well, which are defined by the corresponding source typology. Based on these objects, links are generated, containing a *structural*, a *similarity* or a *Lemma* (Navigli and Ponzetto, 2012) relationship. In this approach, a node is not only created for each source but also for each tag used in the *structural relationship* modeling. Lexical similarities are derived if two nodes have a common lemma in a thesaurus, while string similarities are computed using a suitable metric, in that particular case *N-Grams* (Peterlongo et al., 2005) was used. Similar nodes are merged. Due to this merge, synonyms in user queries can be detected and appropriately handled.

### 3.6. CoreKG

*CoreKG* (Beheshti et al., 2018) contextualizes the metadata in the data catalog. To this end, four features has been identified to constitute this curation service (Beheshti et al., 2017b): *Extraction, Enrichment, Linking* and *Annotation*. The *Extraction* functionality extracts information from the raw data containing natural language, like the names of persons, locations, or organizations. *Enrichment* first provides synonyms and stems from the extracted features by using lexical knowledge bases like *WordNet* (Miller, 1995). These extracted and enriched features then need to be linked to external knowledge bases, like *Wikidata* (Vrandečić, 2012). This enables *CoreKG* to understand, if, for instance, the name of a certain politician was extracted, to link against the corresponding country that politician is active in, i.e., to set it into context. Additionally, users can also annotate the data items.

### 3.7. GOODS

*GOODS* is the internal data lake of Google (Halevy et al., 2016a,b). It is unique compared to all other systems presented since it gathers all its information in a *post-hoc* manner. This means, that the individual teams continue working with their specific tools within their established data silos, while *GOODS* extracts metadata about each dataset by crawling through the corresponding processing logs or storage-system catalogs. The central entity of this data lake is a data set, which can be additionally annotated by users or a special data-stewardship team. These datasets are then connected by a *knowledge graph* (Singhal, 2012) to represent their relationships. Within these relationships, the *dataset containment* enables to split up data sets, as it allows for *bigtable column families* (Chang et al., 2008) to be a data lake entity themselves, along the entire *bigtable*. Due to efficient naming conventions for file paths, *GOODS* can build up *logical clusters*, depending on whether they are regularly, e.g., daily, generated, if they are replicated across different compute centers or if they sharded into smaller data sets. In addition, the data sets are linked by *content similarity* as well. Since the entire data lake contains more than 20 billion data sets with the creation/deletion of 1 billion data sets per day, no pairwise similarity can be performed. Instead, locality-sensitive hash values are generated for individual fields of the data set are generated and compared.

### 3.8. Constance

*Constance* (Hai et al., 2016) is a data lake service, which extracts explicit and implicit metadata from the ingested data, allows semantic annotations and provides derived metadata matching and enrichment for a continuous improvement of the available metadata, and enables inexperienced users to work with simple keyword-based queries by providing a query rewriting engine (Hai et al., 2018). As it is typically done in data lakes, data is ingested in raw format. The next step is to extract as much metadata from it as possible, which is for structured data like *XML* easier since schema definitions can be directly extracted. In the case of semi-structured data, like *JSON* or *CSV* files, a two step process called the *Structural Metadata Discovery* is necessary. First, it is checked, whether or not metadata is either encoded in the raw file itself, like a self-describing spreadsheet or if metadata is encoded in the filename or file path. In a second step, relationships are tried to be discovered during the lifetime of the data lake between the different datasets, for instance, based on the frequencies of join operations. *Semantic Metadata Matching* is provided by a graph model and should use a common ontology. In addition, schemata can be grouped based on their similarity, which is useful in highly heterogeneous data lakes.

## 4. Provenance

One of the generally most important metadata attribute in the context of linked data is provenance (Hartig and Zhao, 2010). Data provenance or the data lineage hereby contains information about the origin of a dataset, e.g., how it was created, by whom, when it was created, etc. There has been an effort by the *W3C* to standardize the representation of provenance information by the use of an OWL2 ontology, as well as a general data model, among other documents to complete their specification called *PROV* (Belhajjame et al., 2013; Missier et al., 2013). Provenance is also becoming increasingly important in science, as it is a natural way to make scientific work more comprehensible and reproducible. This can be exemplified by the adaption of *research objects* (Bechhofer et al., 2010) and *reusable research objects* (Yuan et al., 2018), focusing even more on precise provenance information and repeatability of computational experiments. Apart from this, provenance is considered key in data lakes, to organize, track and link data sets across different transformations and thereby ensure the maintainability of a data lake.

### 4.1. CoreDB and CoreKG

*CoreDB* (Beheshti et al., 2017a) and *CoreKG* (Beheshti et al., 2018) are data lake services with a main emphasis on a comprehensive *REST API*, to organize, index and query data across multiple databases. At the highest level, the main entities of this data lake are data sets, which can be either of type *relational* or of type *NoSQL*. In order to enable simultaneous querying capabilities the CoreDB web service is itself in front of all the other employed services. On this layer, queries are translated between *SQL* and *NoSQL*. A particular focus is lineage tracing of these entities. The recorded provenance is hereby modeled by a directed acyclic graph, where user/roles and entities are nodes while connecting edges represent the interaction. This employed definition is given by the *Temporal Provenance Model* (Beheshti et al., 2012) and can answer, when, from where, by whom, and how a data set was created, read, updated, deleted, or queried.

### 4.2. GOODS

*GOODS* metadata model has a particular focus on provenance (Halevy et al., 2016a,b). In order to build up the provenance graph, production logs are analyzed in a *post-hoc* manner. Then the transitive closure is calculated to determine the linkage between the data sets themselves. Since the data-access events in those logs are extremely high, only a sample is actually calculated and the transient closure is reduced to a limited amount of hops.

### 4.3. Komadu-based provenance auditing

Suriarachchi and Plale (2016a,b) proposed a data lake reference architecture to track data lineage across the lake by utilizing a central provenance collection subsystem. This subsystem enables stream processing of provenance events by providing a suitable *Ingest API* along with a *Query API*. In order to centrally collect provenance and process it, *Komadu* (Suriarachchi et al., 2015) is used. Hereby, distributed components can send provenance information *via RabbitMQ* and web service channels. These single events are then assembled into a global directed acyclic provenance graph, which can be visualized as forward or backward provenance graphs. Using this central subsystem, the need for provenance stitching (Missier et al., 2010) is circumvented.

### 4.4. HPCSerA

A data lake use case is described in the work of Bingert et al. (2021). Here, a user specifies a so-called *job manifest*, which unambiguously describes the job, which should be computed. This includes the actual compute command, the compute environments which are provided by *Singularity* containers (Kurtzer et al., 2017), git repositories which should be cloned and potentially build at run-time, environment variables, user annotations, and most importantly the input and expected output data. This job manifest, written as a *json* document, is then sent to the data lake, which is here represented by a dedicated web application, which is taking control of the actual synchronization with the underlying services, like the high performance compute cluster or the databases. The data lake generates all necessary scripts, which are divided into three phases: i) pre-processing, run, and post-processing. These scripts are submitted to the compute cluster, where within the pre-processing step the compute environment is built on the front-end, and data from a remote S3 storage is staged on a fast parallel file system. Within this step, all possible degrees of freedom, like the input data, or the git commit, are recorded and submitted to the data lake, where it is being indexed. Due to this mechanism, jobs are later on searchable and a provenance graph is automatically created, which connects the artifacts *via* the job manifest as edges to their input or raw data. Due to this recording, as well as the wrapping job manifest, each job is precisely reproducible since one can submit the exact same job without any unknowns.

### 4.5. JUNEAU

*JUNEAU* is build on top of *Jupyter Notebooks*, by replacing its backend and customizing the user interface (Zhang and Ives, 2019). It is therefore specifically targeted at data scientists who are already familiar with Jupyter Notebooks. The main constituents of the data lake are tables, or data frames, of which transformations are tracked. Herefore, the notebook itself is considered to be the workflow, and each executed cell within is a task. The provenance information is captured, when the code within a cell is transmitted to the used *kernel*. Based on this, the notebook is reformatted into a versioned data flow graph, where procedural code is transformed into a declarative form (Ives and Zhang, 2019). Using a modified top-*k* threshold algorithm (Fagin et al., 2003), similar data sets can be found with respect to the individual provenance.

### 4.6. DCPAC

In order to manage automotive sensor data, Robert Bosch GmbH has built a data lake (Dibowski et al., 2020). Although the paper mainly focuses on their extensive DCPAC (Data Catalog, Provenance, Access Control) ontology to build their semantic layer, a dedicated data processing mechanism is provided. Data processing is done using containerized applications, which can access data in the data lake, and either create a new data resource from it or curate existing data sets. The semantic data catalog is updated *via Apache Kafka* messages. Hereby, new data items are integrated and their provenance is automatically recorded.

### 4.7. DataHub

*DataHub* (Bhardwaj et al., 2014) combines a dataset version control system, capable of tracking which operations were performed on which dataset by whom as well as their dependencies, with a hosted platform on top of it. Hereby *DataHub* uses *tables* which contain *records* as their primary entities. *Records* consist of a key, along with any number of typed, named attributes. In the case of completely unstructured data, only the key could then refer to an entire file, in the case of structured or semi-structured files like *XML* or *JSON*, the schema can be (partially) modeled into this *record*. These individual *tables* can then be linked to form *data sets* under specification of the corresponding relationships. The version information of a *table* or *data set* is managed using a *version graph* i.e., a directed acyclic graph where the nodes are data sets and the edges contain provenance information. In order to query multiple versions at a time, a SQL-based query language called *VQL* is provided, which extends SQL about the knowledge that there are different tables for the different versions of a data set.

Along with DataHub, *ProvDB* (Miao et al., 2017; Miao and Deshpande, 2018) is being developed. It incorporates a provenance data model (Chavan et al., 2015) which consists of a *Conceptual Data Model* and a *Physical Property Graph Data Model*. The first model considers a data science project as working directory where all files are either of type *ResultFile, DataFile*, or *ScriptFile*. These files can be further annotated by *properties*, i.e., JSON files. This model is then mapped onto a property graph, where the edges represent the relationship, e.g., parenthood. Provenance ingestion is possible threefold. The first option is to prefix shell commands with *provdb ingest* which then forwards audited information to different specialized *collectors*. Secondly, users can provide annotations. Lastly, there are so-called *File Views*, which allow defining virtual files as a transformation on an existing file. This can be the execution of a script or of an SQL query.

## 5. Support for workflows and automation

Although the first major challenge in building a data lake is the aforementioned metadata management, scaling toward big amounts of data (automated) operation and manageability of the data lake become increasingly important. For example, the extraction of metadata related to data, which is being ingested in a data lake, requires a scalable solution and highly automated processes that best can be integrated into work- or data flows wherever necessary (Mathis, 2017). As in the case of metadata extraction, it is also here sometimes more comfortable to split a complicated analysis up into a workflow consisting of different steps. This has the additional advantage that different parallelization techniques (Pautasso and Alonso, 2006; de Oliveira et al., 2012) can then be applied to improve the scalability of the implemented analysis.

### 5.1. KAYAK

*KAYAK* (Maccioni and Torlone, 2017, 2018) offers so-called *primitives* to analyze newly inserted data in a data lake in an *ad-hoc* manner. KAYAK itself is a layer on top of the file system and offers a user interface for interactions. The respective *primitives* are defined by a workflow of atomic and consistent tasks and can range from inserting or searching for a data set in the data lake, to computing k-means, or performing an outlier analysis. Tasks can be executed either by KAYAK itself, or a third party tool can be triggered, like *Apache Spark* (Zaharia et al., 2016) or *Metanome* (Papenbrock et al., 2015). Furthermore, tasks can be sub-divided into individual steps. By defining a directed acyclic graph, which consists of consecutive dependent primitives, so-called *pipelines* can be constructed with KAYAK. Here, output data is not immediately used as input for a consecutive primitive, but output data is first stored back into the data lake and the corresponding metadata in the data catalog is updated. Users can define a *time-to-action* to specify the maximum time they are willing to wait for a result or preview, or they define a *tolerance*, which specifies the minimal accuracy they demand. A preview is a preliminary result of a step. In order to enable these features, each step has to expose a *confidence* to quantify the uncertainty of the correctness of a preview, and a *cost* function to provide information about the necessary run-time to achieve certain confidence. KAYAK enables the parallel execution of steps by managing dependencies between tasks. These dependencies are modeled as a directed acyclic graph for each primitive. By decomposing these dependency graphs into singular steps, these can be scheduled by a *queue manager*. It enables the asynchronous execution of tasks by utilizing a messaging system to schedule these tasks on a *task executor*, which is typically provided multiple times on a cluster to allow for parallel processing.

### 5.2. Klimatic

*Klimatic* (Skluzacek et al., 2016) integrates over 10,000 different geo-spatial data sets from numerous online repositories. It accesses these data sets *via* HTTP or Globus GridFTP. This done in a manner that allows to capture path-based provenance information and can therein identify relevant data sets based on file extensions, like *NetCDF* or *CSV*. It then pre-processes these heterogeneous data sets to integrate them into this single, homogeneous data lake while ensuring topological, geo-spatial, and user-defined constraints (Elmasri and Navathe, 1994; Cockcroft, 1997; Borges et al., 1999). The pre-processing is done automatically within a three-phase data ingestion pipeline. The first step consists of crawling and scraping, where *Docker* containers are deployed in a scalable pool. These crawlers retrieve a *URL* from a *crawling queue* and then process any data found at that *URL*, while adding newly discovered *URLs* back into the *crawling queue*. Using this approach it is already enough to start with a limited amount of initial repositories, like those of the *National Oceanic and Atmospheric Administration* or the *University Corporation for Atmospheric Research*. After these data sets have been successfully discovered, these are submitted to a *extraction queue*. Elements of this queue are then read by *extractor instances*, and also *Docker* containers which can be elastically deployed. These extract metadata with suitable libraries/tools, like *UK Gemini 2.2*, and then load the extracted metadata into a *PostgreSQL* database. Using these automated processes, a user is for instance able to query for data in a certain range of latitudes and longitudes, and *Klimatic* will estimate the time needed to extract all data from the different data sets within the specified range, and will then provide the user with an integrated data set using focal operations (Shashi and Sanjay, 2003).

## 6. Discussion: Selected use cases and resulting challenges

In this section, two different use cases or user groups, which can be considered to be representative of larger research institutions, are presented. Based on these use cases the previously discussed data lake systems are being analyzed for their applicability in these use cases. In addition, for each section, the presented systems are analyzed and compared to each other. for this, 4 standard criteria are chosen. The *Generality* measures how easily the presented system can be used to cover all kinds of different (research) data. The *Administrative Effort* estimates, how much work is needed to host the system and necessary backend services without actually doing the domain research itself. This is covered by the *Ease of Use*, where the accessibility from a pure user's perspective is analyzed. Lastly, since data lakes are also commonly used in the context of big data, the *Scalability* is a crucial criterion to estimate the worth of deployment. In addition to these four criteria, more topic-specific criteria might be added with regard to the actual focus in the particular section.

### 6.1. User groups

In the following two disparate user groups are presented, which mainly differ in their technical proficiency.

#### 6.1.1. Data scientists

In this use case, mainly data scientists with an above-average technology understanding are interacting with the data lake. Their motivation to use a data lake can come from a big data background, where a massive amount of data should be stored and processed, as well as standardizing processes and their provenance auditing to enhance the reproducibility of their experiments. Hereby, data scientist have the knowledge to work with *SQL, NoSQL*, and graph databases, and to interact with mass storage like *CEPH* (Weil et al., 2006). In order to perform their computations, they rely on *ad-hoc*, local execution of code, e.g., in *Juypter Notebooks* but need to massively scale out their computations in a later stage. Therefore, they need to be able to either work in a cloud environment or on high-performance compute clusters, which are much more cost efficient and are purpose-built for large parallel applications.

#### 6.1.2. Domain scientist

In this use case, mainly domain scientists are using the data lake. It can be assumed, that they are working in a laboratory, or something similar in their respective field. Their motivation to use a data lake is driven by the general necessity of having proper research data management. These users are generally less experienced in dealing with more complicated applications like databases and are generally not required to program large applications or efficient, parallel code for high-performance compute clusters. Data lineage does not only need to be recorded based on digital transformations, i.e., monitoring which artifacts were created by which processes based on which input data, but also along measurements and analysis steps that are happening in laboratories, or comparable. Here, a data lake should be able to manage all experiment data and associated data, e.g., an electronic notebook corresponding to an experiment, and track for instance a sample across the creation and subsequent measurement cycle.

### 6.2. Applicability analysis of the presented data lakes

The following applicability analysis of the previously presented data lakes will be done based on the two provided use cases as well as their perceived administrative effort.

### 6.3. Architecture

#### 6.3.0.1. Zone architecture:

The *Zone Architecture* divides a data lake into different zones to allow for some organization of different data types, which allows for easier automation of repetitive tasks. These zones can be physically implemented on different machines. Users do not necessarily have access to all zones, which means, that they can for instance not directly access raw data, on their own. This entails an administrative effort to serve all users. Additionally, there is no assistance for domain scientists and built-in guidance for reproducible analysis for data scientists by design.

#### 6.3.0.2. Lambda architecture:

The *Lambda Architecture* has some similarities with the zone architecture but has generally a reduced complexity. The rather rigidly coupled batch and speed layers prevent an agile development by scientists but are ideally suited to provide production systems of established workflows while maintaining all raw data sets for later reuse.

#### 6.3.0.3. Lakehouse:

The *Lakehouse* adds a valuable metadata layer on top of an object store and facilitates the advantage of the separation of storage and compute. This, however, entails a limited set of supported file formats and therefore use cases. The completely flat hierarchy and homogeneous usage make this architecture well suited for data scientists and domain scientists alike.

#### 6.3.0.4. FAIR-DO based architecture:

The *FAIR Digital Object-based Architecture* offers a fine-grained refinement based on the types which also have a clear abstraction, increasing the general comprehensibility. The administrative effort is decreased, since new data types are derived from existing ones and general data lake functionalities only need to be implemented once for the parent objects, afterwards they can be reused in user space. The flat architecture does not intrinsically restrict access and offers a homogeneous interface across all stages. This allows to implement a customized but homogeneous user interfaces for domain researchers covering the entire life cycle of a certain experiment. Meanwhile, can data scientists work with the well-known abstraction of objects they are familiar with from the object-oriented programming paradigm. The possibility to globally enforce data governance based on the typed attributes of these *FAIR Digital Objects* is well suited to integrate different data sources or silos into a single research data management system.

#### 6.3.0.5. Functional and maturity-based architectures:

The classification in either *functional*-based, *maturity*-based, or hybrid data lakes undermines the importance to sort data based on their refinement, i.e., on the degree of processing they were subjected to, while also stretching the importance to formulate activities on the data lake as functions. This has the advantage of a high standardization which eases the administrative overhead while guaranteeing minimal data quality and adherence to institutional policies. It is hard to distinguish here between domain researchers and data scientists since it is not clear how actual implementations of these concepts would respect the different needs of those user groups.

#### 6.3.0.6. Qualitative comparison:

Looking at the four presented architectures one can do a qualitative comparison as shown in Table 1. As discussed, the zone architecture has the highest complexity of all four of them, therefore lacking in administrative effort and ease of use, but it has a high generality. The Lambda architecture reduces the complexity compared to zone architecture, and is, therefore, easier to maintain and use, but is not as versatile applicable, since it mainly serves production systems with established workflows. Similar arguments can be found for the lakehouse, which can only support limited file formats. The FAIR Digital Object-based architecture has a high generality since it can wrap any existing file. Always offering Digital Objects to interact with is comfortable for the users but requires more administrative work, particularly at the beginning. One can also see that all architectures fulfill the general requirement for a data lake to be scalable.

**Table 1:** Comparing the four presented architectures.

**Architecture**	**Generality**	**Administrative effort**	**Ease of use**	**Scalability**
Zone	+	–	–	+
Lambda	0	+	+	+
Lakehouse	0	+	+	+
FAIR-DO	+	0	+	+

Putting the presented architectures into the context of the overall evolution of data lakes which were in their first years mostly realized using Hadoop clusters and the associated software stack (Khine and Wang, 2018), one can see a clear development toward more abstracted systems. The first level of abstraction was proposed by Functional-based architectures, which can also be mapped on Zone architectures by associating a certain functionality with a certain zone. This idea was greatly advanced by the FAIR-DO-based Architecture where the users don't see the actual system they are working on, but only trigger the execution of predefined functions using a REST API. This approach will lower the entry barrier, particularly for domain researchers, while restricting the general risk of a loss of consistency across the data lake. The general idea to organize the data in the data lake regarding their pedigree of subjected processing has clearly convinced, as it is also a fundamental part of the newer architectures, i.e., the FAIR-DO based Architecture, and the Maturity-based architecture. Since the lambda architecture only offers the serving layer, this is also true here. Although there was the strive in data lakes to separate storage and compute, the importance of storage performance becomes more important in the recent developments around lakehouses. Here, in future work, one should include active storage into the entire concept. Promising ideas are shown by Chakraborty et al. (2022), which extends the capabilities of a Ceph cluster.

#### 6.3.1. Metadata models

##### 6.3.1.1. Data vault

Although *Data Vaults* may seem old at first glance, they actually offer a generic and flexible way of modeling diverse data sets into a single model. However, designing a proper *Data Vault* is very challenging and requires deep knowledge about data modeling in general as well as about the usage of databases. Therefore, this model rather seems to be more suited for data scientists than domain researchers, while the administrative overhead depends on the used system.

##### 6.3.1.2. GEMMS

The *Generic and Extensible Metadata Management System* was particularly designed for research data. The concept of a data unit seems straightforward, however, semantic annotations are only possible with a suitable ontology. Although this increases the quality of the resulting metadata catalog, resulting in challenges like ontology merging by administrators and the effort of domain researchers to get their vocabulary into these ontologies is a drawback. There was also no example provided, of how this model can be used in conjunction with unstructured data.

##### 6.3.1.3. MEDAL and goldMEDAL

Also in these models, global metadata like ontologies and thesauri are necessary. The improved version of *goldMEDAL* seems matured, as it only uses straight forward concepts as *data entities, processes, links*, and *groupings*. More unintelligible concepts like representations have been dropped. The presented implementation used *Neo4J*, which is within the defined realm of data scientists. An open challenge seems to be the integration of fully automated processes with adherence to global governance to ensure data quality and to guide inexperienced users through the data lake.

##### 6.3.1.4. CODAL

*CODAL* was purpose-built for textual data, it, therefore, lacks the capacity to scale to generic use cases. The combination of a *Data Vault* with a graph database along with the usage of *XML* documents seems only suited for experienced users, i.e., data scientists. Combining these two models seems powerful for the specific use case, however, entails a corresponding administrative overhead.

##### 6.3.1.5. Network-based models

This model is also a graph-based model, which aims to integrate heterogeneous and particularly unstructured data into a single research data management system. The notion of objects, represented as nodes, offers the necessary versatility, to adapt to different use cases. The required definition of the corresponding source typology might not be easily implementable for domain scientists, but the overhead of including experienced administrators for the initial setup seems reasonable. However, the powerful relationship modeling using *structural, similarity* and *Lemma* relationships will introduce quite some maintenance overhead. This model, therefore, seems more appropriate for well-experienced data scientists who can ensure correct implementation and operation.

##### 6.3.1.6. CoreKG

This data model was discussed in detail for the case of data containing natural language. The proposed model and presented workflow to implement this model are very convincing with the big restriction, that it is only meaningful and implementable for text documents containing natural language. Once the workflow is set up, it seems useful for data scientists as well as domain researchers.

##### 6.3.1.7. GOODS

*GOODS* builds up a data catalog in a *post-hoc* manner. It creates *data sets* which are enriched with information within logs and user annotations. These *data sets* are then connected by a knowledge graph to represent their relationships. Although the idea to build up a data lake in a *post-hoc* manner seems very promising for any larger institution, it is connected with large challenges. Each log format and naming convention, also on the file path level, needs to be understood by the data lake. This requires for instance domain researchers to strictly follow global data governance, which usually requires additional auditing. Also on an administrative site, such a setup is as difficult to implement as it is compelling to have. Accessing all systems from one central data lake also comes with certain security risks which need to be addressed, increasing the complexity even more. Therefore, as impressive as this internal data lake of Google is, it is most likely out of reach for other, much smaller research institutions.

##### 6.3.1.8. Constance

The presented metadata model in *Constance* was applied to structured and semi-structured data, where metadata was automatically extracted from the raw data itself or in the full file path. This approach lacks the ability to upload an associated metadata file. This could be done by a domain researcher who uploads an electronic lab book containing all metadata of a certain experiment. If this metadata needs to be indexed to enable a semantic search over it, such a mechanism needs to be provided. Furthermore, the usage of ontologies enables semantic metadata matching on the one side, while on the other side this might be hard to implement. Problems here are, that rather data scientists are trained to use them compared to domain researchers and that the broader a data lake becomes, the more likely the introduction of an additional ontology becomes, which then might require complicated merges of ontologies (Noy and Musen, 2003; Hitzler et al., 2005). Therefore, this approach seems more feasible for data scientists who are operating on a restricted set of data sources.

##### 6.3.1.9. Qualitative comparison

In Table 2 a qualitative comparison of the eight discussed models is provided. In addition to the previously used criteria, *Similar Dataset Exploration* and *Semantic Enrichment* is added. The first one describes the possibility to find new data in a data lake that is similar to data a user already has found. This is for instance for statistical analysis like machine learning important, to be able to increase the input data set. Semantic Enrichment describes the possibility to, ideally continuously, add semantic information to data in the data lake to improve the findability. Implementing and using a data vault on top of a SQL or NoSQL database requires a manageable amount of time. In addition, it is scalable, one can describe generic entities and their relations, and allows for evolution over time, therefore enabling not a continuous but a discrete semantic enrichment. GEMMS was not yet by default extended to support any file type, and the use of ontologies has certain disadvantages, like ontology merging and user consultation. It is also not completely clear how similar data sets can be found and how a continuous semantic enrichment can be performed. MEDAL is a rather complicated model, with a steep learning curve. Relying on Representation which is derived by transformation will probably limit the usage scenarios. Similarity links allow for easy dataset exploration and allow for semantic enrichment. The revised version goldMEDAL improves, compared to MEDAL, usability by dropping the complicated Representation and Transformation relationship and reducing it to simpler Processes. CODAL was purpose-built for textual data, and thus lacks generality. In addition, relying on a filesystem limits scalability. Updating semantic metadata in an xml file, however, allows for continuous semantic enrichment, and connecting all data entities with nodes representing relationships allows for a good dataset exploration. The network-based models can describe generic data, but however, the more complicated notion of source typologies decreases the ease of use. Using N-Grams to compute similarities, similar data sets can be detected. Structural metadata can be randomly added to allow for semantic enrichment. CoreKG is again a purpose-built metadata model for textual data, therefore it is not generalizable. setting up the full curation service requires some administrative effort, but offers afterwards an easy and powerful model. The enrichment and linking service enable continuous data curation and exploration. GOODS requires that necessary metadata is encoded in log files or in storage-system catalogs, which limits the generality. The administrative effort here is enormous, however, the ease of use for the users is great since no change to their data silos and employed techniques is necessary. The capability to scale across dozens or hundreds of data centers is leading and the integration into an existing knowledge graph enables similar dataset explorations. The evaluation of semantic enrichment is difficult due to the high velocity of the data. Constance offers a generic metadata model, however, the structural metadata discovery did not explicitly include unstructured data like images. The query rewriting engine eases the use drastically and offers similar dataset exploration and semantic enrichment.

**Table 2:** Comparing the nine presented metadata models.

**Model**	**Generality**	**Administrative effort**	**Ease of use**	**Scalability**	**Similar dataset Exploration**	**Semantic enrichment**
Data Vault	+	0	0	0	+	0
GEMMS	0	0	–	0	–	0
MEDAL	0	0	–	0	+	+
goldMEDAL	0	0	0	0	+	+
CODAL	-	0	0	-	+	+
Network Models	+	0	–	0	+	+
CoreKG	-	–	+	0	+	+
GOODS	0	–	+	+	+	0
Constance	0	0	+	0	+	+

To summarize, the discussed metadata models offer diverse approaches for data modeling. However, there are common patterns across these models. All of these models have an atomic entity around which the entire modeling evolves. In order to harmonize these different atomic entities, in some models called objects, ontologies are commonly utilized. This increases the entry barrier, particularly for domain researchers, and can always lead to the necessity to perform ontology merging. These models also always employed a method to model relationships between their atomic entities, or aggregations of them. As a general observation, one can state that a proper metadata model for data lakes has to offer some means to describe, also semantically, its entities on its own, as well as their relationship toward each other. This has led to the simultaneous usage of different database systems, i.e., SQL, NoSQL, and Graph databases, within a single data lake, which introduced the challenge to query these different systems with a single user query. More powerful query-rewriting engines and/or suitable meta-languages which support the integration of semantic meaning within this process is one of the challenges metadata modeling in data lakes is currently facing. In addition, semantic metadata extraction from particularly unstructured data, like images, is also a key challenge to improve the usability and adoption of data lakes.

#### 6.3.2. Provenance

##### 6.3.2.1. CoreDB(KG)

The employed *Temporal Provenance Model* is suitable for data scientists and domain researchers, although it is not a widely used standard. However, no details about the technical implementation and the versatility are given. Therefore, no final assessment of the actual applicability is possible.

##### 6.3.2.2. GOODS

Apart from the already discussed challenges of employing a *post-hoc* analysis to populate a central data lake, an additional challenge arises when using this approach to gather provenance information: The used analysis software needs to write suitable logs. Since this is not the case for all scientific software, this approach is hard to implement for domain researchers, while data scientists might be able to cope with that issue when using self-written code or wrappers around existing software suits. Interestingly, only *GOODS* calculates the transitive closure of the provenance graph, which seems very useful.

##### 6.3.2.3. Komadu

This data lake implementation offers a dedicated *Ingest API* which uses a *RabbitMQ* messaging queue, to retrieve lineage information about the performed processing steps. The transparent assembly of these singular tasks to a global provenance graph is comfortable and useful. As in the *GOODS* discussion, data scientists can use custom build software or write wrappers around existing ones to utilize the messaging system. Domain researchers will probably have a hard time when their scientific software suit does not support this kind of provenance auditing.

##### 6.3.2.4. HPCSerA

Here, users are required to describe their analysis they want to run in a *job manifest*. This job is then executed on an HPC system. By using *Singularity* containers and enabling the dynamic build and integration of arbitrary git commits, this integrates well with a typical HPC workflow. These systems are often used by data scientists, which benefit here from transparent provenance auditing of completely generic jobs and the ability to re-run a previous analysis to reproduce the results. This mechanism can also be extended for better Cloud support, however, there is a lack of an *ad-hoc* analysis with a similar provenance auditing, which might be important for domain researchers.

##### 6.3.2.5. JUNEAU

This modification for *Jupyter Notebook* offers an *ad-hoc* experience for users, who are working with *Python* and with tabularized data. Since *Jupyter Notebook* is broadly utilized by data scientists and domain researchers alike, it is generally suited for both groups. However, this approach only works for tabularized data and only supports *Python* which is limiting the possible use cases. In addition, this presented data lake implementation fell short of detailed data and metadata handling and mainly focused on *ad-hoc* processing. It remains unclear, how well this implementation is able to serve as a central research data management system for a variety of data sources and user groups.

##### 6.3.2.6. DCPAC

Data lineage in *DCPAC* is recorded by custom build containers which send messages to *Apache Kafka*. This approach requires users to containerize their applications and implement a messaging mechanism. This is a suitable method for data scientists but is challenging for domain researchers. particularly for domain researchers, it would be necessary to check the messages for quality and consistency.

##### 6.3.2.7. DataHub

This platform which offers a data set version control system is a good solution for all researchers. The representation of the provenance within a *version graph* is interesting and straightforward. In addition, the possibility to use *ProvDB* offers more detailed modeling capabilities on the basis of files. The ingestion of the provenance data, however, is not generic enough. Using the shell command, will not offer a suitable depth, for instance, to offer full reproducibility, while on the other hand, the *file views* are only suitable for data scientists familiar with *SQL*. The third provided option, i.e., user annotations, is very error prone and is therefore unsuited as a guideline for good scientific practice.

##### 6.3.2.8. Qualitative comparison

In Table 3 a qualitative comparison of the seven discussed models is provided. In addition to the previously used criteria, *Reproducibility* is added. This specifies, whether based on the gathered provenance information, a result can always be reproduced, which becomes increasingly relevant for scientific publications. CoreDB/CoreKG offers a RestAPI and thereby an easy-to-use interface and the distinction between the types SQL and NoSQL offers a great generality. However, the employed Temporal Provenance Model is not an established standard and is not aimed to guarantee reproducibility, but rather comprehensibility. GOODS relies on production logs, based on which heuristics are used to calculate the provenance graph. It is aimed for scalability and efficiency, not for reproducibility. Komadu relies on a RabbitMQ messaging, it is therefore not generally applicable. The provided RestAPI is a useful user interface. However, reproducibility relies on the quality of the messages, thus it is depending on the analytics job running, and is independent of the data lake itself. The data lake which uses HPCSerA can execute arbitrary scripts and it offers a RestAPI to work with. Its strength lies in its transparent lineage auditing on an HPC system by using Job Manifests. By storing and linking all entities, every artifact can be reproduced. The inclusion of HPC systems makes this setup very scalable. JUNEAU is extremely user-friendly by building directly on top of the well-known Jupyter Notebooks. Therefore it lacks generality and scalability since it depends on data frames and is limited to the resources of a single notebook. The transparent lineage recording during the submission of the code in a cell to the kernel allows reproducibility. DCPAC works on arbitrary data, however the usage of an extensive ontologies requires users to familiarize with it. The usage of Docker containers is scalable, and offers a fair reproducibility. DataHub can deal with any kind of data and offers a comfortable graphical user interface. The, although limited, provenance information in conjunction with the version control of the data allows for decent reproducibility. In conclusion, while all previously discussed data lake implementations share the common idea of building a global provenance graph, there exists a wealth of different provenance models and auditing methods. In the future, data lakes should focus more on established models for provenance representation, to enhance interoperability. Furthermore, from the fact that each data lake implementation found a unique provenance auditing approach, it becomes clear that in each case a specific processing mechanism was in mind, like an HPC system in HPCSerA or a Jupyter Notebook in JUNEAU. This means, that not a single data lake offered provenance auditing capabilities over the entire life cycle of a data-driven project for generic applications. A future challenge here is, to support provenance auditing in *ad-hoc* analytics, like within a Jupyter Notebook, as well as in larger computations that run in a Cloud or HPC environment and integrate this into a homogeneous, global provenance graph, ideally in a reproducible manner. These single tasks need then to be linked to workflows, with the same provenance granularity. A similar challenge here is to support generic applications, without relying on a built-in messaging or logging functionality.

**Table 3:** Comparing the seven provenance models.

**Implementation**	**Generality**	**Administrative effort**	**Ease of use**	**Scalability**	**Reproducibility**
CoreDB/CoreKG	+	0	+	+	0
GOODS	0	–	+	+	–
Komadu	-	0	+	0	0
HPCSerA	+	0	+	+	+
JUNEAU	–	0	+	–	+
DCPAC	+	0	0	+	0
DataHub	+	0	+	0	+

#### 6.3.3. Support for workflows and automation

##### 6.3.3.1. KAYAK

*KAYAK* offers a very sophisticated mechanism to implement parallelizable and automatable workflows. The decomposition of an abstract workflow into *tasks* which can then be combined to *primitives* and finally can be chained to entire *pipelines*, requires some prior knowledge. Although powerful, it is rather suited for data scientists, and not so much for domain researchers, since the above decomposition of established scientific software suits into these entities is not straightforward. Furthermore, although the idea of a *tolerance* and a *time-to-action* is very useful on a data lake, this is only suitable for a subset of methods that are iterative by nature. From the viewpoint of a generic scientific application, this might simply add additional overhead and increase the entry barrier. Therefore, this well-designed data lake is mostly suitable for experienced data scientists.

##### 6.3.3.2. Klimatic

The automated processing capabilities of *Klimatic* are based on *Docker* containers. The generic approach to split up a pipeline into distinct stages which are linked by dedicated queues can be adopted to serve other use cases as well. Although it was only used for data set exploration within this particular implementation, also data analysis pipelines could be set up using this approach. This would require building containers that are pushing back their output into a certain queue, or the current data lake implementation can be extended to offer a generic wrapping method that accepts arbitrary containers and then orchestrates the communication with the queuing system. One can see, how this data lake can be extended to also serve domain researchers from other sciences as well.

##### 6.3.3.3. Qualitative comparison

In Table 4 a qualitative comparison of the two discussed processing concepts is provided. KAYAK has a scalable and parallelizable approach to executing a workflow. Since user-defined Primitives are supported, it is also very generic. In addition, the user interface on top of the filesystem eases the use, however, the entire execution model with its depth of options requires some time to familiarize with it. Klimatic presents a specific use case and it is not completely clear how generalizable the general approach is. Setting all queues and ingestion pipelines up for the first time requires some administrative effort but is, therefore, more comfortable for users to use. The usage of remote Docker hosts to serve multiple workers which get their jobs from a queue is also very scalable.

**Table 4:** Comparing the two workflow and automation tools.

**Implementation**	**Generality**	**Administrative effort**	**Ease of use**	**Scalability**
KAYAK	+	0	0	+
Klimatic	0	–	+	+

In conclusion, there is only a limited amount of work focusing on workflows and automation for processes on data lakes. The challenge here is to incorporate a scalable back-end to support the compute-intensive operations associated with big data. Using portable containers is a well-suited approach. Future developments, however, would largely benefit from a modularized approach allowing to integrate different back-ends in a suitable manner, i.e., to have native support for individual machines, as well as for Cloud and HPC environments. This extends explicitly to the employed workflow engines, which should similarly prevent a lock-in effect, as envisioned by *CWL* (Amstutz et al., 2016).

## 7. Summary and outlook

This paper presents and summarizes the most relevant papers connected to data lakes and analyzes the past developments to identify future challenges. This is done with a particular focus on the applicability for larger research institutions which are characterized by diverse user groups, which are for simplicity represented by domain researchers and data scientists within this paper.

One can see in Section 2, that there is a trend toward an abstraction of the underlying systems. This allows to conceptually model the data life-cycle and increases the usability by defining a certain functionality within this life-cycle. Furthermore, by only exposing predefined functions to users the consistency of the data lake can be ensured, even when used by inexperienced users. To increase the general user group of a data lake, it is important, that the metadata model is similarly easy to use and yet generic enough to be suitable for diverse use cases.

In Section 3 it was seen, that there is the general need to model relationships between some atomic data lake entities. In addition, these atomic entities also need to be described by semantic metadata, which will be more intuitive, particularly for domain researchers. The most important challenge here is, to find a metadata model, which offers a low entry barrier for domain researchers to fill in their data, but offers a enough depth to for experienced users to utilize the sophisticated methods for special use cases, as they are presented.

By analyzing the papers which are presented in Section 4 it becomes clear, that there is the open quest to develop a uniform provenance auditing mechanism which is able to capture homogeneous lineage information along the entire project life-cycle, reaching from first *ad-hoc* scripts to large-scale parallel applications.

Also, in Section 5 there is a clear trend toward containerized applications to enable processing on data lakes. The advantages are many-fold, reaching from portability to an increased reproducibility. The provided mechanisms to allow for parallel and asynchronous executions are convincing. The next future challenge can be identified to enable these methods to use different back-ends, reaching from single systems, to public or private clouds, and HPC systems.

The concluding analysis in Section 6 took more different criteria into account and compared the presented data lake systems based on the criteria. Here one could see, that there is no single data lake system, which fulfills all requirements. Instead, most of the systems are to some extend purpose built systems, which are compromising in some aspects to better exceed at other. In addition, two different user groups from the view point of a larger research institution were defined in Section 6.1. Based on these user groups a more subjective analysis was done, with the purpose to motivate the general accessibility of this user group to certain data lake implementation. This might be useful in order to improve systems to attract more user, wich is the largest challenge currently in data lake development. However, it would have also the largest benefit, to get diverse user groups excited of the idea of data lakes. This will lead to an increased influx of new (meta-) data and methods, and the integration of previously siloed data will enable novel analysis which have not been possible before.

## Author contributions

HN: writing original draft, analysis, and interpretation of existing literature. PW: writing original draft, writing review, and supervision. Both authors reviewed the results and approved the final version of the manuscript.

## Funding

We gratefully acknowledge funding by the Niedersächsisches Vorab funding line of the Volkswagen Foundation and Nationales Hochleistungsrechnen (NHR), a network of eight computing centers in Germany to provide computing capacity and promote methodological skills.

## Conflict of interest

The authors declare that the research was conducted in the absence of any commercial or financial relationships that could be construed as a potential conflict of interest.

## Publisher's note

All claims expressed in this article are solely those of the authors and do not necessarily represent those of their affiliated organizations, or those of the publisher, the editors and the reviewers. Any product that may be evaluated in this article, or claim that may be made by its manufacturer, is not guaranteed or endorsed by the publisher.

## Footnotes

^1^https://flume.apache.org/.

^2^https://orc.apache.org/.

## References

[B1] Amstutz P. Crusoe M. R. Tijaníc N. (2016). Common Workflow Language. v1. 0. Available online at: https://www.commonwl.org/v1.0/Workflow.html (accessed August 07, 2022).

[B2] Armbrust M. Das T. Sun L. Yavuz B. Zhu S. Murthy M.. (2020). Delta lake: high-performance acid table storage over cloud object stores. Proc. VLDB Endowment 13, 3411–3424. 10.14778/3415478.3415560

[B3] Armbrust M. Ghodsi A. Xin R. Zaharia M. (2021). Lakehouse: a new generation of open platforms that unify data warehousing and advanced analytics, in Proceedings of CIDR.

[B4] Armbrust M. Xin R. S. Lian C. Huai Y. Liu D. Bradley J. K.. (2015). Spark sql: relational data processing in spark, in Proceedings of the 2015 ACM SIGMOD International Conference on Management of Data (Melbourne, VIC), 1383–1394.

[B5] Aundhkar A. Guja S. (2021). A review on enterprise data lake solutions. J. Sci. Technol. 6, 11–14. 10.46243/jst.2021.v6.i04.pp11-14

[B6] Batyuk A. Voityshyn V. (2016). Apache storm based on topology for real-time processing of streaming data from social networks, in 2016 IEEE First International Conference on Data Stream Mining and Processing (DSMP) (Lviv: IEEE), 345–349.

[B7] Bechhofer S. De Roure D. Gamble M. Goble C. Buchan I. (2010). Research objects: toward exchange and reuse of digital knowledge. Nat. Preced. 10.1038/npre.2010.4626.1

[B8] Beheshti A. Benatallah B. Nouri R. Chhieng V. M. Xiong H. Zhao X. (2017a). Coredb: a data lake service, in Proceedings of the 2017 ACM on Conference on Information and Knowledge Management (Singapore), 2451–2454.

[B9] Beheshti A. Benatallah B. Nouri R. Tabebordbar A. (2018). Corekg: a knowledge lake service. Proc. VLDB Endowment 11, 1942–1945. 10.14778/3229863.3236230

[B10] Beheshti S.-M.-R. Motahari-Nezhad H. R. Benatallah B. (2012). Temporal provenance model (TPM): model and query language. arXiv preprint arXiv:1211.5009. 10.48550/arXiv.1211.5009

[B11] Beheshti S.-M.-R. Tabebordbar A. Benatallah B. Nouri R. (2017b). On automating basic data curation tasks, in Proceedings of the 26th International Conference on World Wide Web Companion (Perth, WA), 165–169.

[B12] Belhajjame K. B'Far R. Cheney J. Coppens S. Cresswell S. Gil Y.. (2013). Prov-dm: The prov data model. Technical Report.

[B13] Bhardwaj A. Bhattacherjee S. Chavan A. Deshpande A. Elmore A. J. Madden S.. (2014). Datahub: collaborative data science and dataset version management at scale. arXiv preprint arXiv:1409.0798. 10.48550/arXiv.1409.0798

[B14] Bingert S. Köhler C. Nolte H. Alamgir W. (2021). An API to include HPC resources in workflow systems, in INFOCOMP 2021, The Eleventh International Conference on Advanced Communications and Computation (Porto), ed C. -P. Rückemann, 15–20.

[B15] Borges K. A. Laender A. H. Davis C. A. Jr (1999). Spatial data integrity constraints in object oriented geographic data modeling, in Proceedings of the 7th ACM International Symposium on Advances in Geographic Information Systems (Kansas City, MO), 1–6.

[B16] Borthakur D. (2007). The hadoop distributed file system: architecture and design. Hadoop Project Website 11, 21.

[B17] Chakraborty J. Jimenez I. Rodriguez S. A. Uta A. LeFevre J. Maltzahn C. (2022). Skyhook: towards an arrow-native storage system. arXiv preprint arXiv:2204.06074. 10.1109/CCGrid54584.2022.00017

[B18] Chang F. Dean J. Ghemawat S. Hsieh W. C. Wallach D. A. Burrows M.. (2008). Bigtable: a distributed storage system for structured data. ACM Trans. Comput. Syst. 26, 1–26. 10.1145/1365815.1365816

[B19] Chavan A. Huang S. Deshpande A. Elmore A. Madden S. Parameswaran A. (2015). Towards a unified query language for provenance and versioning, in 7th USENIX Workshop on the Theory and Practice of Provenance (TaPP 15) (Edinburgh).

[B20] Cockcroft S. (1997). A taxonomy of spatial data integrity constraints. Geoinformatica 1, 327–343. 10.1023/A:1009754327059

[B21] de Oliveira D. Ogasawara E. Oca na K. Bai ao F. Mattoso M. (2012). An adaptive parallel execution strategy for cloud-based scientific workflows. Concurrency Comput. 24, 1531–1550. 10.1002/cpe.1880

[B22] Dean J. Ghemawat S. (2008). Mapreduce: simplified data processing on large clusters. Commun. ACM. 51, 107–113. 10.1145/1327452.1327492

[B23] Devlin B. A. Murphy P. T. (1988). An architecture for a business and information system. IBM Syst. J. 27, 60–80. 10.1147/sj.271.0060

[B24] Diamantini C. Giudice P. L. Musarella L. Potena D. Storti E. Ursino D. (2018). A new metadata model to uniformly handle heterogeneous data lake sources, in European Conference on Advances in Databases and Information Systems (Nicosia: Springer), 165–177.

[B25] Dibowski H. Schmid S. Svetashova Y. Henson C. Tran T. (2020). Using semantic technologies to manage a data lake: data catalog, provenance and access control, in SSWS@ ISWC (Athen), 65–80.

[B26] Dixon J. (2010). Pentaho, Hadoop, and Data Lakes. Available online at: https://jamesdixon.wordpress.com/2010/10/14/pentaho-hadoop-and-data-lakes/ (accessed April 22, 2022).

[B27] Elmasri R. Navathe S. (1994). Fundamentals of database systems.

[B28] El-Sappagh S. H. A. Hendawi A. M. A. El Bastawissy A. H. (2011). A proposed model for data warehouse ETL processes. J. King Saud Univer. Comput. Inf. Sci. 23, 91–104. 10.1016/j.jksuci.2011.05.00533190464

[B29] Fagin R. Lotem A. Naor M. (2003). Optimal aggregation algorithms for middleware. J. Comput. Syst. Sci. 66, 614–656. 10.1016/S0022-0000(03)00026-6

[B30] Giebler C. Gröger C. Hoos E. Eichler R. Schwarz H. Mitschang B. (2021). The data lake architecture framework: a foundation for building a comprehensive data lake architecture, in Proceedings der 19. Fachtagung für Datenbanksysteme für Business, Technologie und Web (BTW 2021).

[B31] Giebler C. Gröger C. Hoos E. Schwarz H. Mitschang B. (2019). Modeling data lakes with data vault: practical experiences, assessment, and lessons learned, in International Conference on Conceptual Modeling (Salvador: Springer), 63–77.

[B32] Giebler C. Gröger C. Hoos E. Schwarz H. Mitschang B. (2020). A zone reference model for enterprise-grade data lake management, in Proceedings of the 24th IEEE Enterprise Computing Conference (EDOC 2020) (Eindhoven: IEEE).

[B33] Golec D. (2019). Data lake architecture for a banking data model, in ENTRENOVA-ENTerprise REsearch InNOVAtion, Vol. 5 (Zagreb), 112–116.

[B34] Gorelik A. (2019). The Enterprise Big Data Lake: Delivering the Promise of Big Data and Data Science. Sebastopol, CA: O'Reilly Media.

[B35] Hai R. Geisler S. Quix C. (2016). Constance: an intelligent data lake system, in Proceedings of the 2016 International Conference on Management of Data (San Francisco, CA), 2097–2100.

[B36] Hai R. Quix C. Jarke M. (2021). Data lake concept and systems: a survey. arXiv preprint arXiv:2106.09592. 10.48550/arXiv.2106.09592

[B37] Hai R. Quix C. Zhou C. (2018). Query rewriting for heterogeneous data lakes, in European Conference on Advances in Databases and Information Systems (Budapest: Springer), 35–49.

[B38] Halevy A. Korn F. Noy N. F. Olston C. Polyzotis N. Roy S.. (2016a). Goods: organizing google's datasets, in Proceedings of the 2016 International Conference on Management of Data (San Francisco, CA), 795–806.

[B39] Halevy A. Y. Korn F. Noy N. F. Olston C. Polyzotis N. Roy S.. (2016b). Managing google's data lake: an overview of the goods system. IEEE Data Eng. Bull. 39, 5–14. 10.1145/2882903.2903730

[B40] Hartig O. Zhao J. (2010). Publishing and consuming provenance metadata on the web of linked data, in International Provenance and Annotation Workshop (Troy: Springer), 78–90.

[B41] Hasani Z. Kon-Popovska M. Velinov G. (2014). Lambda architecture for real time big data analytic, in ICT Innovations (Ohrid), 133–143.

[B42] Hitzler P. Krötzsch M. Ehrig M. Sure Y. (2005). What is ontology merging? in American Association for Artificial Intelligence (Palo Alto, CA: AAAI Press), 4.

[B43] Hukkeri T. S. Kanoria V. Shetty J. (2020). A study of enterprise data lake solutions, in International Research Journal of Engineering and Technology (IRJET), Vol. 7 (Tamilnadu).

[B44] Inmon B. (2016). Data Lake Architecture: Designing the Data Lake and Avoiding the Garbage Dump. Basking Ridge, NJ: Technics Publications.

[B45] Inmon W. H. (2005). Building the Data Warehouse. Indianapolis, IN: John Wiley & Sons.

[B46] Ives Z. G. Zhang Y. (2019). Dataset relationship management, in Proceedings of Conference on Innovative Database Systems Research (CIDR 19) (Monterey, CA).

[B47] Khine P. P. Wang Z. S. (2018). Data lake: a new ideology in big data era. ITM Web Conf. 17, 03025. 10.1051/itmconf/20181703025

[B48] Kurtzer G. M. Sochat V. Bauer M. W. (2017). Singularity: Scientific containers for mobility of compute. PLoS ONE 12, e0177459. 10.1371/journal.pone.0177459 28494014 PMC5426675

[B49] Li J. (2014). Design of real-time data analysis system based on impala, in 2014 IEEE Workshop on Advanced Research and Technology in Industry Applications (WARTIA) (Ottawa, ON: IEEE), 934–936.

[B50] Lindstedt D. Graziano K. (2011). Super Charge Your Data Warehouse: Invaluable Data Modeling Rules to Implement Your Data Vault. North Charleston, CA: CreateSpace.

[B51] Maccioni A. Torlone R. (2017). Crossing the finish line faster when paddling the data lake with kayak. Proc. VLDB Endowment 10, 1853–1856. 10.14778/3137765.3137792

[B52] Maccioni A. Torlone R. (2018). Kayak: a framework for just-in-time data preparation in a data lake, in International Conference on Advanced Information Systems Engineering (Tallinn: Springer), 474–489.

[B53] Madera C. Laurent A. (2016). The next information architecture evolution: the data lake wave, in Proceedings of the 8th International Conference on Management of Digital Ecosystems (Biarritz), 174–180.

[B54] Madsen M. (2015). How to Build an Enterprise Data Lake: Important Considerations Before Jumping in. San Mateo, CA: Third Nature Inc.

[B55] Mathis C. (2017). Data lakes. Datenbank Spektrum 17, 289–293. 10.1007/s13222-017-0272-7

[B56] Miao H. Chavan A. Deshpande A. (2017). Provdb: Lifecycle management of collaborative analysis workflows, in Proceedings of the 2nd Workshop on Human-in-the-Loop Data Analytics (Chicago, IL), 1–6.

[B57] Miao H. Deshpande A. (2018). Provdb: provenance-enabled lifecycle management of collaborative data analysis workflows. IEEE Data Eng. Bull. 41, 26–38. 10.1145/3077257.3077267

[B58] Miller G. A. (1995). Wordnet: a lexical database for english. Commun. ACM. 38, 39–41. 10.1145/219717.219748

[B59] Miloslavskaya N. Tolstoy A. (2016). Big data, fast data and data lake concepts. Procedia Comput. Sci. 88, 300–305. 10.1016/j.procs.2016.07.439

[B60] Missier P. Belhajjame K. Cheney J. (2013). The W3C PROV family of specifications for modelling provenance metadata, in Proceedings of the 16th International Conference on Extending Database Technology (Genoa), 773–776.

[B61] Missier P. Ludäscher B. Bowers S. Dey S. Sarkar A. Shrestha B.. (2010). Linking multiple workflow provenance traces for interoperable collaborative science, in The 5th Workshop on Workflows in Support of Large-Scale Science (New Orleans, LA: IEEE), 1–8.

[B62] Munappy A. R. Bosch J. Olsson H. H. (2020). Data pipeline management in practice: challenges and opportunities, in Product-Focused Software Process Improvement, ed Morisio M. Torchiano M. Jedlitschka A. (Cham: Springer International Publishing), 168–184.

[B63] Munshi A. A. Mohamed Y. A.-R. I. (2018). Data lake lambda architecture for smart grids big data analytics. IEEE Access 6, 40463–40471. 10.1109/ACCESS.2018.2858256

[B64] Navigli R. Ponzetto S. P. (2012). Babelnet: The automatic construction, evaluation and application of a wide-coverage multilingual semantic network. Artif. Intell. 193, 217–250. 10.1016/j.artint.2012.07.001

[B65] Nogueira I. D. Romdhane M. Darmont J. (2018). Modeling data lake metadata with a data vault, in Proceedings of the 22nd International Database Engineering and Applications Symposium (Villa San Giovanni), 253–261.

[B66] Nolte H. Wieder P. (2022). Realising data-centric scientific workflows with provenance-capturing on data lakes. Data Intell. 4, 426–438. 10.1162/dint_a_00141

[B67] Noy N. F. Musen M. A. (2003). The prompt suite: interactive tools for ontology merging and mapping. Int. J. Hum. Comput. Stud. 59, 983–1024. 10.1016/j.ijhcs.2003.08.002

[B68] Oram A. (2015). Managing the Data Lake: Moving to Big Data Analysis. Sebastopol, CA: O'Reilly Media.

[B69] Papenbrock T. Bergmann T. Finke M. Zwiener J. Naumann F. (2015). Data profiling with metanome. Proc. VLDB Endowment 8, 1860–1863. 10.14778/2824032.2824086

[B70] Patel P. Wood G. Diaz A. (2017). Data lake governance best practices, in The DZone Guide to Big Data-Data Science and Advanced Analytics, Vol. 4 (Durham, NC), 6–7.

[B71] Pautasso C. Alonso G. (2006). Parallel computing patterns for grid workflows, in 2006 Workshop on Workflows in Support of Large-Scale Science (Paris: IEEE), 1–10.

[B72] Pérez-Arteaga P. F. Castellanos C. C. Castro H. Correal D. Guzmán L. A. Denneulin Y. (2018). Cost comparison of lambda architecture implementations for transportation analytics using public cloud software as a service, in Special Session on Software Engineering for Service and Cloud Computing (Porto), 855–862.

[B73] Peterlongo P. Pisanti N. Boyer F. Sagot M.-F. (2005). Lossless filter for finding long multiple approximate repetitions using a new data structure, the bi-factor array, in International Symposium on String Processing and Information Retrieval (Buenos Aires: Springer), 179–190.

[B74] Quix C. Hai R. Vatov I. (2016). Gemms: a generic and extensible metadata management system for data lakes, in CAiSE Forum, Vol. 129 (Ljubljana).

[B75] Ramakrishnan R. Sridharan B. Douceur J. R. Kasturi P. Krishnamachari-Sampath B. Krishnamoorthy K.. (2017). Azure data lake store: a hyperscale distributed file service for big data analytics, in Proceedings of the 2017 ACM International Conference on Management of Data (Chicago, IL), 51–63.

[B76] Ravat F. Zhao Y. (2019). Data lakes: trends and perspectives, in International Conference on Database and Expert Systems Applications (Linz: Springer), 304–313.

[B77] Sawadogo P. Darmont J. (2021). On data lake architectures and metadata management. J. Intell. Inf. Syst. 56, 97–120. 10.1007/s10844-020-00608-7

[B78] Sawadogo P. N. Scholly E. Favre C. Ferey E. Loudcher S. Darmont J. (2019). Metadata systems for data lakes: models and features, in European Conference on Advances in Databases and Information Systems (Bled: Springer), 440–451.

[B79] Scholly E. Sawadogo P. Liu P. Espinosa-Oviedo J. A. Favre C. Loudcher S.. (2021). Coining goldmedal: a new contribution to data lake generic metadata modeling. arXiv preprint arXiv:2103.13155. 10.48550/arXiv.2103.13155

[B80] Sethi R. Traverso M. Sundstrom D. Phillips D. Xie W. Sun Y.. (2019). Presto: Sql on everything, in 2019 IEEE 35th International Conference on Data Engineering (ICDE) (Macao: IEEE), 1802–1813.

[B81] Sharma B. (2018). Architecting Data Lakes: Data Management Architectures for Advanced Business Use Cases. Sebastopol, CA: O'Reilly Media.

[B82] Shashi S. Sanjay C. (2003). Spatial databases: A Tour. Upper Saddle River, NJ: Prentice Hall.

[B83] Singhal A. (2012). Introducing the knowledge graph: things, not strings. Off. Google Blog 5, 16.

[B84] Skluzacek T. J. Chard K. Foster I. (2016). Klimatic: a virtual data lake for harvesting and distribution of geospatial data, in 2016 1st Joint International Workshop on Parallel Data Storage and data Intensive Scalable Computing Systems (PDSW-DISCS) (Salt Lake City, UT: IEEE), 31–36.

[B85] Suriarachchi I. Plale B. (2016a). Crossing analytics systems: a case for integrated provenance in data lakes, in 2016 IEEE 12th International Conference on e-Science (e-Science) (Baltimore, MD: IEEE), 349–354.

[B86] Suriarachchi I. Plale B. (2016b). Provenance as essential infrastructure for data lakes, in International Provenance and Annotation Workshop (McLean, VA: Springer), 178–182.

[B87] Suriarachchi I. Zhou Q. Plale B. (2015). Komadu: a capture and visualization system for scientific data provenance. J. Open Res. Software 3, e4. 10.5334/jors.bq

[B88] Terrizzano I. G. Schwarz P. M. Roth M. Colino J. E. (2015). Data wrangling: the challenging yourney from the wild to the lake, in CIDR (Asilomar).

[B89] Thusoo A. Sarma J. S. Jain N. Shao Z. Chakka P. Anthony S.. (2009). Hive: a warehousing solution over a map-reduce framework. Proc. VLDB Endowment 2, 1626–1629. 10.14778/1687553.1687609

[B90] Villari M. Celesti A. Fazio M. Puliafito A. (2014). Alljoyn lambda: an architecture for the management of smart environments in iot, in 2014 International Conference on Smart Computing Workshops (Hong Kong: IEEE), 9–14.

[B91] Vohra D. (2016). Apache parquet. In Practical Hadoop Ecosystem. New York, NY: Springer.

[B92] Vrandečić D. (2012). Wikidata: a new platform for collaborative data collection, in Proceedings of the 21st International Conference on World Wide Web (Lyon), 1063–1064.

[B93] Walker C. Alrehamy H. (2015). Personal data lake with data gravity pull, in 2015 IEEE Fifth International Conference on Big Data and Cloud Computing (Dalian: IEEE), 160–167.

[B94] Warren J. Marz N. (2015). Big Data: *Principles and Best Practices of Scalable Realtime Data Systems*. Shelter Island, NY: Simon and Schuster.

[B95] Weil S. A. Brandt S. A. Miller E. L. Long D. D. Maltzahn C. (2006). Ceph: a scalable, high-performance distributed file system, in Proceedings of the 7th Symposium on Operating Systems Design and Implementation (Seattle, WA), 307–320.

[B96] Yuan Z. Ton That D. H. Kothari S. Fils G. Malik T.. (2018). Utilizing provenance in reusable research objects. Informatics, 5, 14. 10.3390/informatics5010014

[B97] Zaharia M. Chowdhury M. Franklin M. J. Shenker S. Stoica I. (2010). Spark: cluster computing with working sets, in 2nd USENIX Workshop on Hot Topics in Cloud Computing (HotCloud 10) (New York, NY).

[B98] Zaharia M. Xin R. S. Wendell P. Das T. Armbrust M. Dave A.. (2016). Apache spark: a unified engine for big data processing. Commun. ACM. 59, 56–65. 10.1145/2934664

[B99] Zhang Y. Ives Z. G. (2019). Juneau: data lake management for jupyter. Proc. VLDB Endowment 12, 3352095. 10.14778/3352063.3352095

[B100] Zikopoulos P. (2015). Big Data Beyond the Hype: A Guide to Conversations for Today's Data Center. New York, NY; Chicago, IL; San Francisco, CA; Athens; Athens; London; Madrid; Mexico City; Milan; New Delhi; Singapore; Sydney, NSW; Toronto, ON: McGraw-Hill Education.

